# Können Mesh-Vernebler die prähospitale Aerosoltherapie verbessern? Eine In-vitro-Studie an simulierten Notfallpatient*innen mit Atemnot

**DOI:** 10.1007/s00101-022-01183-y

**Published:** 2022-08-17

**Authors:** M. Otto, Y. Kropp, L. Kummer, M. Thiel, C. Tsagogiorgas

**Affiliations:** 1 Klinik für Anästhesiologie und Operative Intensivmedizin, Universitätsmedizin Mannheim, Medizinische Fakultät Mannheim, Universität Heidelberg, Theodor-Kutzer-Ufer 1–3, 68167 Mannheim, Deutschland; 2 Klinik für Anästhesiologie, Universitätsklinikum Heidelberg, Heidelberg, Deutschland; 3 Klinik für Anästhesie und Intensivmedizin, St. Elisabethenkrankenhaus Frankfurt, Frankfurt am Main, Deutschland

**Keywords:** Notfallmedizin, Rettungsdienst, Medikamentenvernebelung, Verneblertyp, Atemnot, Emergency medicine, Emergency medical service, Inhalation therapy, Nebulizers, Dyspnea

## Abstract

**Hintergrund:**

Medikamentenvernebler im Rettungsdienst sollten eine hohe Vernebelungsleistung haben, um schnell eine therapeutische Wirkstoffkonzentration des vernebelten Medikaments zu erreichen. Eine Umfrage im süddeutschen Rettungsdienst zeigte allerdings, dass fast ausschließlich die wenig effizienten Jet-Vernebler zum Einsatz kommen.

**Ziel der Arbeit:**

Ziel der vorliegenden In-vitro-Studie war es herauszufinden, ob der Einsatz von Mesh-Verneblern die prähospitale Aerosoltherapie verbessern könnte.

**Material und Methoden:**

Die Vernebelungsleistung eines Jet-Verneblers (Cirrus™ 2, Fa. Intersurgical®) und 2 mobil einsatzbarer Mesh-Vernebler (Aerogen Solo®, Fa. Aerogen Limited, M‑Neb® mobile, NEBU-TEC International med. Produkte Eike Kern GmbH) wurde in einem In-vitro-Modell spontan atmender Notfallpatient*innen mit 4 unterschiedlichen Atemmustern bei verschiedenen Sauerstoffflussraten getestet.

**Ergebnisse:**

Die Mesh-Vernebler zeigten im Vergleich zum Jet-Vernebler eine signifikant höhere Verneblungsleistung und Salbutamol-Filterdeposition, wobei der M‑Neb® mobile die höchsten Werte für Leistung und Deposition erreichte. Der Sauerstofffluss hatte den größten Einfluss auf die Leistung des Jet-Verneblers, wirkte sich aber kaum auf die Mesh-Vernebler aus. Die Deposition wurde zudem stark vom Atemmuster beeinflusst.

**Diskussion:**

Der Einsatz von Mesh-Verneblern mit hoher Verneblungsleistung konnte in einem In-vitro-Modell die Aerosoltherapie von prähospitalen Notfallpatient*innen verbessern. Sie waren dem Jet-Vernebler in Bezug auf die Verneblungsleistung und die Lungendeposition überlegen und ermöglichten zudem eine bedarfsangepasste Sauerstofftherapie. Die höchste Medikamentendeposition wurde bei den tachypnoischen Patient*innen erreicht, welche in der praktischen Anwendung auch am meisten von einem erhöhten Medikamentenspiegel profitieren würden.

**Zusatzmaterial online:**

Zusätzliche Informationen sind in der Online-Version dieses Artikels (10.1007/s00101-022-01183-y) enthalten (weitere Tabellen).

## Abstract (en)

**Background:**

Nebulizers used to treat prehospital emergency patients should provide a high output efficiency to achieve a fast onset of therapeutic drug effects while remaining unaffected by the presence of supplementary oxygen flow or the patient’s breathing pattern. On the other hand, nebulizer performance is directly influenced by differences in device design, gas flow and patients’ breathing patterns. Several studies from emergency departments were able to demonstrate an improvement in patient outcome when using a mesh nebulizer instead of a jet nebulizer. Data or bench studies regarding prehospital care are non-existent.

**Objective:**

The aim of the present in vitro study was to evaluate which type of aerosol generator would best address the requirements of a prehospital adult emergency patient suffering from respiratory distress.

**Material and methods:**

We evaluated the performance of a jet nebulizer (Cirrus™ 2, Intersurgical®) and two mesh nebulizers (Aerogen Solo® with USB controller, Aerogen Limited and M‑Neb® mobile, NEBU-TEC International med. Produkte Eike Kern GmbH) with the possibility of portable operation in an in vitro model of a spontaneously breathing adult emergency patient. One physiological and three pathological breathing patterns (distressed breathing pattern as well as stable and acute exacerbated chronic obstructive pulmonary disease) were simulated. Nebulizer output and salbutamol lung deposition were measured at different oxygen flow rates using a face mask as the delivery interface.

**Results:**

The mesh nebulizers produced a significantly higher aerosol output when compared to the jet nebulizer. The M‑Neb® mobile was able to significantly exceed the output of the Aerogen Solo®. Oxygen flow had the largest influence on the output of the jet nebulizer but hardly affected the mesh nebulizers. After a nebulization time of 10 min the M‑Neb® mobile also achieved the highest total salbutamol lung deposition (*P* < 0.001). Aerosol drug deposition was therefore mainly determined by the nebulizer’s drug output per unit time. The deposition could not be improved using a spacer but was strongly influenced by the simulated emergency patients’ breathing pattern.

**Conclusion:**

The use of mesh nebulizers might have the potential to improve the aerosol therapy of prehospital emergency patients. In general, mesh nebulizers seem to be superior to jet nebulizers regarding aerosol output per unit time and total lung deposition. The present data suggest that aerosol output and drug deposition to the collection filter in this simulated setting are closely connected and crucial for total salbutamol deposition, as the deposition could not be improved by adding a spacer. Aerosol drug deposition in simulated emergency patients’ lungs is therefore mainly determined by the nebulizer’s drug output per unit time.

The level of oxygen flow used had the largest influence on the output of the jet nebulizer but hardly affected the output of the tested mesh nebulizers. Mesh nebulizers could therefore enable a demand-adapted oxygen therapy due to their consistent performance despite the presence of oxygen flow. A high respiratory rate was associated with a high drug deposition, which is clinically desirable in the treatment of patients in respiratory distress; however, drug underdosing must also be expected in the treatment of bradypneic patients. Further clinical studies must prove whether our findings also apply to the treatment of real prehospital emergency patients.

## Hinführung zum Thema

Die Behandlung von Notfallpatient*innen mittels vernebelter Medikamente hat einen festen Stellenwert in der prähospitalen Notfallmedizin. Allerdings existieren keinerlei Empfehlungen, welcher Verneblertyp hierbei genutzt werden soll. In einer kürzlich durchgeführten Befragung des süddeutschen Rettungsdienstpersonals konnte gezeigt werden, dass in der Praxis fast ausschließlich Jet-Vernebler zur Aerosoltherapie eingesetzt werden, die eigentlich in puncto Leistung überlegenen Mesh-Vernebler wurden dagegen kaum eingesetzt. Für Notfallambulanzen konnte aber bereits eine Verbesserung des Patienten-Outcomes gezeigt werden, wenn zur inhalativen Therapie von Notfallpatient*innen Mesh-Vernebler verwendet werden. Daten oder Grundlagenstudien zur prähospitalen Patientenversorgung existieren bis dato noch nicht.

## Hintergrund und Fragestellung

Zu den wichtigsten Anforderungen an Medikamentenvernebler, die zur Aerosoltherapie von prähospitalen Notfallpatient*innen eingesetzt werden, gehören eine hohe Verneblungsleistung und eine hohe Medikamentendeposition in der Lunge, um ein schnelles Einsetzen der therapeutischen Wirkung zu erreichen [12]. Das verwendete Verneblersystem, die individuellen Atemmuster der Patient*innen und auch eine parallel durchgeführte Sauerstofftherapie beeinflussen die Verneblerleistung und Lungendeposition allerdings maßgeblich [2, 16, 31].

Zwar gibt es Leitlinien, die vorgeben, wann im Rettungsdienst die Vernebelungstherapie eingesetzt werden soll, allerdings wird nicht spezifiziert, welcher Aerosolgenerator verwendet werden soll [29]. Dies wäre wichtig, da eine kürzlich durchgeführte Umfrage im süddeutschen Rettungsdienst gezeigt hat, dass zur Aerosoltherapie von Notfallpatient*innen fast ausschließlich Jet-Vernebler zum Einsatz kommen [24]. Diese erreichen bei Spontanatmenden aber eine deutlich geringere Medikamentendeposition als Mesh-Vernebler [1, 22]. Studien aus Notaufnahmen konnten bereits eine Verbesserung des Patienten-Outcome demonstrieren, wenn Mesh-Vernebler verwendet wurden [13, 17].

Auch benötigen Notfallpatient*innen mit Atemnot häufig Sauerstoff [8]. Eine hohe Frischgasflussrate kann die Aerosoldeposition bei spontan atmenden Patient*innen aber erheblich beeinflussen [2]. Auch unterscheiden sich die Atemmuster der zugrunde liegenden Erkrankung hinsichtlich Tidalvolumen, Atemfrequenz, Inspirationszeit und I:E-Verhältnis [30]. Diese Parameter haben ebenfalls Einfluss auf die pulmonale Medikamentendeposition [5].

Die Empfehlung eines bestimmten Verneblertyps wird dadurch erschwert, dass Grundlagenstudien zur Verwendung im Rettungsdienst fehlen. Daher wurden in einem In-vitro-Modell eines simulierten, spontan atmenden Notfallpatienten die Verneblungsleistung und die Medikamentendeposition eines Jet-Verneblers (Cirrus™2, Intersurgical®, Sankt Augustin, Deutschland) und zweier mobil nutzbarer Mesh-Vernebler (Aerogen® Solo mit USB-Controller, Aerogen Limited, Galway, Irland und M‑Neb® mobile, NEBU-TEC International med. Produkte Eike Kern GmbH, Elsenfeld, Deutschland) bei unterschiedlichen Atemmustern und unterschiedlichen supplementären Sauerstoffflussraten verglichen. Als Notfallmedikament wurde exemplarisch Salbutamol gewählt.

## Studiendesign und Untersuchungsmethode

### In-vitro-Modell eines simulierten, spontan atmenden Notfallpatienten mit und ohne Atemnot

**Figure 1:**
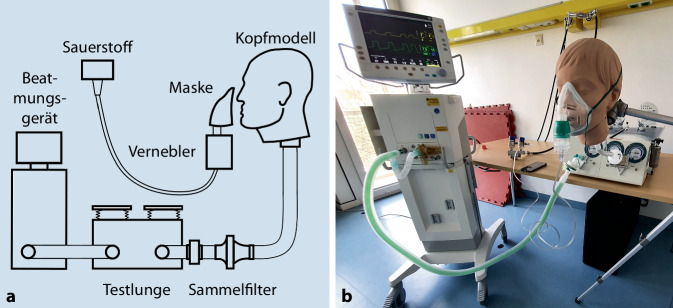

Grundlage des In-vitro-Modells war eine duale Testlunge für Erwachsene (Fa. Michigan Instruments, Kentwood, MI, USA), die auch spontane Atmung simulieren kann. Diese wurde an ein Beatmungsgerät (Engström Carestation; Fa. GE Healthcare, Chicago, IL, USA) angeschlossen. Ein Kopfmodell eines Erwachsenen (HSM‑A; Fa. Michigan Instruments) wurde mit der Testlunge verbunden. Zum Sammeln und zum späteren Quantifizieren des durch die Vernebler produzierten Salbutamolaerosols wurde ein Geräteschutzfilter (Respirgard II; Fa. Vyaire Medical Inc., Mettawa, IL, USA), wie er in gängigen In-vitro-Modellen zur Aerosolforschung verwendet wird, zwischen dem Kopf und der Testlunge platziert, wie in Abb. 1 dargestellt [15].

### Atemmuster

Es wurden 4 unterschiedliche Atemmuster simuliert (Tab. 1). Für normale Atmung und Atemnot wurden in der Literatur bereits etablierte Muster übernommen [7, 27]. Um eine chronisch obstruktive Lungenerkrankung (COPD) zu simulieren, wurden 2 von Bauer et al. vorgeschlagene Atemmuster verwendet [5]. Bei der COPD-Exazerbation wurde die Atemfrequenz an die aktuellen Datenlage angepasst [5, 19].

**Table 1:** 

	Atemparameter			
Atemmuster	Tidalvolumen (ml)	Atemfrequenz (Atemzüge/min)	I:E Verhältnis	Inspirationszeit (s)
Normal	500	15	1:2	1,3
Atemnot	750	30	1:1	1,0
COPD, stabil	650	10	1:3,6	1,3
COPD, exazerbiert	300	24	1:0,7	2,1

### Vernebler und Maske

**Figure 2:**
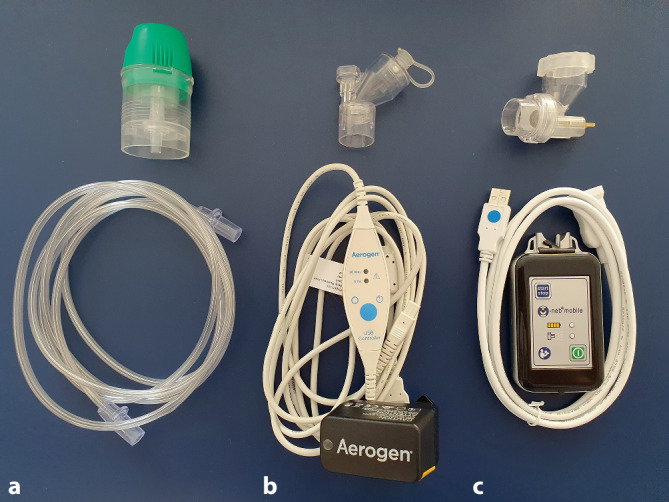

Getestet wurden mobil verwendbare Vernebler, die in Deutschland verfügbar sind. Dies würde eine Verwendung sowohl an der Einsatzstelle, auf dem Weg zum Fahrzeug und im Fahrzeug selbst ermöglichen. Diese Kriterien erfüllten 2 Verneblertypen, darunter ein Jet-Vernebler und 2 Mesh-Vernebler, in Abb. 2 dargestellt.

**Figure 3:**
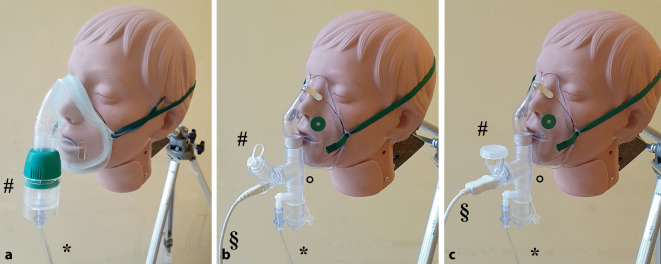

Der Jet-Vernebler (Cirrus 2; Fa. Intersurgical, Sankt Augustin) wurde an eine Gesichtsmaske für Erwachsene (Intersurgical EcoLiteMask; Fa. Intersurgical, Sankt Augustin) angebracht (Abb. 3a). Der zum Betrieb des Jet-Verneblers obligate Flow wurde von einer tragbaren 2‑l-Sauerstoffflasche bereitgestellt. Die Verneblungsleistung wurde bei Flussraten von 6 l/min und 12 l/min getestet. Auf die Testung bei einer Flussrate von 1 l/min wurde verzichtet, da der Jet-Vernebler hier kein Aerosol generiert.

Der erste Mesh-Vernebler (Aerogen Solo mit USB-Controller; Fa. Aerogen Limited, Galway, Irland) wurde über eine USB-Steuereinheit betrieben. Das Steuergerät wurde über eine USB-Powerbank mit Strom versorgt. Zum Betrieb der Mesh-Vernebler ist grundsätzlich kein Frischgasfluss notwendig. Der Vernebler wurde bei Flussraten von 1 l/min (Herstellerempfehlung), 6 l/min und 12 l/min mit Gesichtsmaske (Fa. Salter Labs, SunMed, Grand Rapids, MI, USA), wie in Abb. 3b dargestellt, und in der Kombination mit Gesichtsmaske und eigens für den Vernebler entwickeltes Spacer-System (Aerogen Ultra; Fa. Aerogen Limited) getestet.

**Figure 4:**
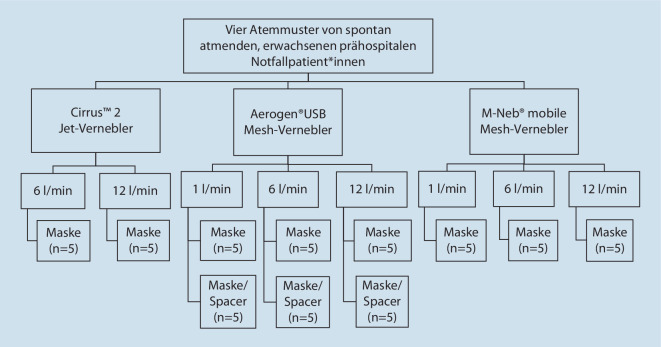

Der zweite Mesh-Vernebler (M-Neb mobile; Fa. NEBU-TEC International med. Produkte Eike Kern GmbH, Elsenfeld) wurde von einer Steuereinheit mit internem Akku betrieben. Der Vernebler wurde an eine Gesichtsmaske (Fa. Salter Labs, SunMed, Grand Rapids, MI, USA) angeschlossen (Abb. 3c) und bei Sauerstoffflüssen von 1 l/min, 6 l/min und 12 l/min getestet. Das Studienprotokoll ist in Abb. 4 dargestellt.

### Ablauf der Experimente

Zur besseren Filterextraktion und Konzentrationsanalyse wurde eine 2,5-mg/ml-Salbutamollösung (Konzentration_Salbutamol_) (Fa. GSK, London, GB) verwendet. Zur Bestimmung des Volumens in den Verneblerkammern wurde dieselbe gravimetrische Methode verwendet, wie von El Taoum et al. vorgeschlagen, welche die Annahme voraussetzt, dass 1 ml Salbutamollösung 1 g wiegt [18].

Zu Beginn jedes Testlaufs wurden die leeren Verneblerkammern mit einer Präzisionswaage (Scaltec SBC 31; Fa. Scaltec Instruments GmbH, Göttingen, Deutschland) mit einer Ablesegenauigkeit von 0,0001 g gewogen (Gewicht_leer_). Anschließend wurde die Kammer jedes Verneblertyps mittels einer kalibrierten 1000-µl-Pipette (Fa. BRAND, Wertheim, Deutschland) mit 5 ml der Salbutamollösung befüllt und anschließend erneut gewogen (Gewicht_gefüllt_). Dann wurde der Vernebler zusammengebaut und an die Maske angeschlossen. Die Verneblungsdauer aller Vernebler betrug 10 min. Nach Abschluss der Vernebelung wurde die Kammer erneut gewogen (Gewicht_Ende_). Wenn ein Vernebler vor Ablauf der 10 min leer war, wurde die Zeit gestoppt und die Kammer gewogen, erneut gefüllt und dann abermals gewogen. Dann wurde die Verneblung fortgesetzt.

### Verarbeitung der Salbutamolproben

Nach Ende der Verneblung wurden 5 ml destilliertes Wasser mit einer 1000-µl-Pipette (Fa. BRAND, Wertheim) in die Verneblerkammer gegeben. Die Kammer wurde gewogen (Gewicht_Ende_ +5) und dann verschlossen, bevor sie 2 min lang gevortext wurde, um mögliche Salbutamolrückstände von den Kammerwänden abzuwaschen. Die Menge an Salbutamol im fertigen Eluat wurde als der Anteil des in der Verneblerkammer verbliebenen Salbutamols angesehen.

Für die Extraktion des Salbutamols aus den Filtern wurde dieser aus dem Filtergehäuse entfernt, in ein 50-ml-Röhrchen gegeben und mittels Pipette 10 ml destilliertes Wasser hinzugefügt. Das Röhrchen wurde verschlossen und 2 min lang gevortext. Die Menge an Salbutamol im Eluat wurde als die Menge des in den Atemwegen und der Lunge abgelagerten Salbutamols betrachtet.

### Analyse und Quantifizierung des Salbutamols

Mit einem Spektrofotometer (Fa. BioTek Epoch2 microplate reader, Bad Friedrichshall, Deutschland) wurde die Salbutamolkonzentration im Eluat des Filters (Konzentration_Filter_) und der Kammer (Konzentration_Kammer_) bei einer Wellenlänge von 276 nm gemessen [14]. Zur Berechnung der Kalibrierungskurven mittels linearer Regressionsanalyse wurde IBM SPSS Statistics 25 verwendet. Die Kalibrierungskurven waren über den gesamten Bereich linear.

### Berechnung der Verneblerleistung

Die Verneblerleistung wurde wie folgt berechnet:

$$\text{Verneblerleistung}\,\left(\frac{\mathrm{ml}}{10\,\text{min}}\right)=\text{Volumen}_{\text{bef{\"u}llt}}-\text{Volumen}_{\text{Ende}}$$

Unter der Annahme, dass 1 ml der Salbutamollösung 1 g wiegt, folgte:

$$\text{Verneblerleistung}\left(\frac{\text{ml}}{10\,\text{min}}\right)=\text{Gewicht}_{\text{gef{\"u}llt}}-\text{Gewicht}_{\text{Ende}}$$

### Berechnung der Salbutamol-Filterkonzentration

Mit der Formel *Masse* = *Konzentration* × *Volumen*, wobei Volumen (Menge Spüllösung) und Konzentration (Ergebnis der Fotometeruntersuchung) bekannt waren, wurde die Salbutamol-Filtermasse (M_Filter_) berechnet

$${\text{Masse}_{\text{Filter}}}\left(\text{mg}\right)=\text{Konzentration}_{\text{Filter}\,}\times 10\,\text{ml}$$

### Statistische Auswertung

Die statistische Analyse erfolgte mit IBM SPSS Statistics 25. Die Daten zeigten eine Normalverteilung (*p* > 0,05 für Kolmogorov-Smirnov- und Shapiro-Wilk-Test). Zum Vergleich der kumulierten deponierten Salbutamolmenge und der Verneblungsleistung wurde eine Welch-ANOVA durchgeführt, da Varianzhomogenität nicht gegeben war (Levene-Test *p* < 0,05). Für die Post-hoc-Analyse wurde ein Games-Howell-Test verwendet. Alle Variablen wurden mit Mittelwert ± SD angegeben. Die statistische Signifikanz wurde auf *p* < 0,05 festgelegt.

## Ergebnisse

### Verneblerleistung

**Figure 5:**
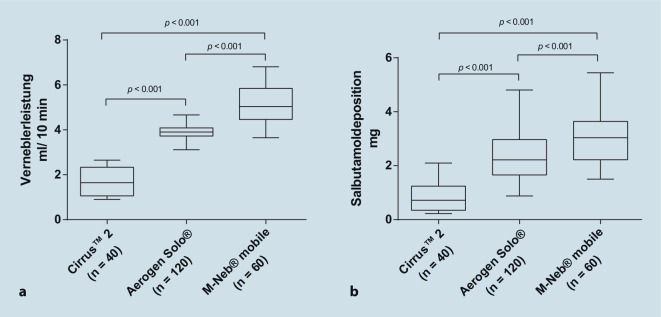

Nach einer Vernebelungszeit von 10 min zeigte der M‑Neb mobile im Vergleich zu allen anderen getesteten Verneblern den signifikant höchsten kumulierte Aerosolausstoß pro Zeit (5,13 ml ± 0,81 ml). Der Aerogen Solo erzeugte den zweithöchsten Aerosolausstoß (3,90 ml ± 0,29 ml); dieser war deutlich höher als der des Cirrus 2 Jet-Verneblers (1,71 ml ± 0,67 ml). Alle Ergebnisse sind in Abb. 5a dargestellt. Die Leistung der einzelnen Setups ist in Tabelle A des Zusatzmaterial online dargestellt.

#### Einfluss des Sauerstoffflusses auf die Verneblerleistung

Verglichen mit einer Flussrate von 6 l/min produzierte der Cirrus 2 Jet-Vernebler mehr als die doppelte Menge an Aerosol, wenn er mit einer Sauerstoffflussrate von 12 l/min betrieben wurde (1,05 ml ± 0,06 ml vs. 2,36 ml ± 0,18 ml; *P* < 0,001). Bei einem Flow von 1 l/min zeigte der Aerogen-Solo-Vernebler ohne Spacer einen signifikant niedrigeren Ausstoß im Vergleich zu 6 l/min (3,6 ml ± 0,26 ml vs. 3,99 ml ± 0,3 ml; *P* < 0,003) und 12 l/min (3,6 ml ± 0,26 ml vs. 3,99 ml ± 0,17 ml; *P* < 0,001). Die Aerosolausstöße des M‑Neb mobile und des Aerogen Solo mit angebrachtem Spacer wurden durch den Flow nicht signifikant beeinflusst.

### Salbutamol-Filterdeposition

Nach einer Vernebelungszeit von 10 min erreichte der M‑Neb mobile im Vergleich zu allen anderen getesteten Verneblern die signifikant höchste kumulierte Salbutamol-Filterdeposition (3,01 mg ± 0,87 mg; *p* < 0,001 vs. alle), Abb. 5b. Der Aerogen Solo erzeugte die zweithöchste (2,38 mg ± 0,87 mg), der Jet-Vernebler die niedrigste Filterdeposition (0,79 mg ± 0,50 mg). Die Verwendung eines Spacers hatte keinen signifikanten Einfluss auf die Deposition. Die Deposition der einzelnen Setups wird in Tabelle B des Zusatzmaterial online dargestellt.

#### Einfluss des Sauerstoffflusses auf die Salbutamol-Filterdeposition

Die Filterdeposition des Cirrus-2-Jet-Verneblers war bei einer Sauerstoffflussrate von 12 l/min mehr als 3‑mal so hoch wie bei 6 l/min (1,22 mg ± 0,34 mg vs. 0,36 mg ± 0,09 mg; *P* < 0,001). Bei einem Flow von 1 l/min erreichte der M‑Neb mobile im Vergleich zum Aerogen Solo eine signifikant höhere Filterdeposition; bei 6 l/min bzw. 12 l/min zeigte sich kein statistisch signifikanter Unterschied.

#### Einfluss des Atemmusters auf die Salbutamol-Filterdeposition

**Figure 6:**
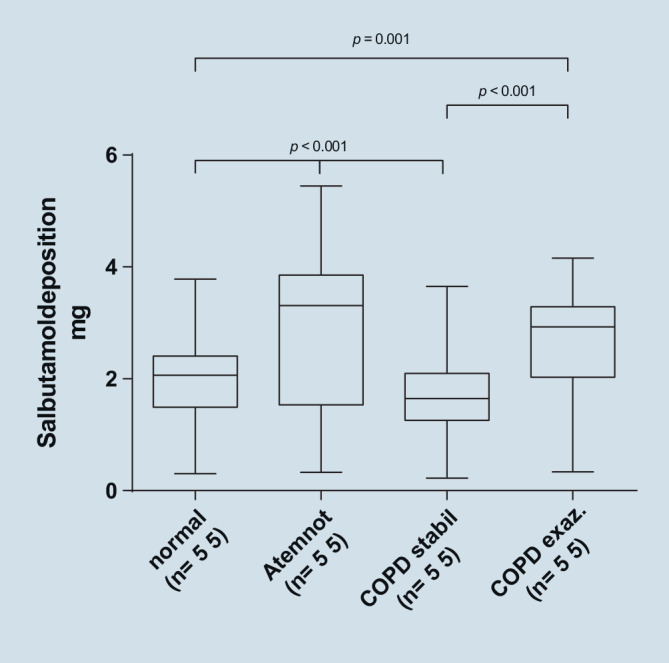

Das Atemmuster hatte einen signifikanten Einfluss auf die Deposition von Salbutamol (*p* < 0,001). Die größten kumulierten Salbutamolmengen wurden bei der Simulation von Atemnot (2,84 mg ± 1,32 mg) und exazerbierter COPD (2,62 mg ± 1,01 mg) gefunden, wie in Abb. 6 dargestellt.

## Diskussion

### Verneblerleistung

Wie erwartet produzierten beide Mesh-Vernebler deutlich mehr Aerosol pro Zeit als der Jet-Vernebler [1, 16]. Der M‑Neb mobile konnte den Aerogen Solo noch übertreffen, zeigte aber auch eine größere Standardabweichung. Dieser Leistungsvorteil gegenüber Jet-Vernebler macht Mesh-Vernebler attraktiv für den Einsatz im prähospitalen Rettungsdienst [12]. Bei der Behandlung von Atemnot ist ein rasches Einsetzen der therapeutischen Medikamentenwirkung entscheidend, da Beschwerden so schneller gelindert, eine Beatmung möglicherweise abgewendet oder der anschließende Aufenthalt in der Notfallambulanz verkürzt werden könnten [17]. Trotz der hier demonstrierten Limitationen ist der Jet-Vernebler aber der mit Abstand am häufigsten verwendete Verneblertyp im süddeutschen Rettungsdienst [24].

Manche Notfallpatient*innen benötigen Sauerstoff. Der Flow sollte die Verneblerleistung aber nicht negativ beeinflussen [8, 12]. Studien konnten bereits zeigen, dass der Flow besonders die Leistung von Jet-Verneblern beeinflusst [21]. Auch die vorliegende Arbeit zeigt, dass die Leistung des Cirrus 2 mehr als doppelt so hoch war, wenn er mit einem Flow von 12 l/min anstelle von 6 l/min betrieben wurde. Allerdings würde ein höherer Flow klinisch nicht zu einer Verbesserung des Sauerstoffangebots führen, da die F_I_O_2_ bei der Verwendung von Gesichtsmasken ohne Reservoir kaum über 42–47 % steigt [10]. Auch die Verwendung niedrigerer Flussraten bei geringem O_2_-Bedarf ist nicht möglich, da dies die Leistung des Jet-Verneblers drastisch vermindert. Eine individuelle, an den Bedarf angepasste Sauerstofftherapie ist daher mit Jet-Verneblern kaum möglich. Neben der Verwendung von speziellen, in Deutschland unbekannten Systemen (Neb-U-Mask®) könnte der parallele Einsatz einer High-Flow-Nasenbrille die F_I_O_2_ zusätzlich erhöhen, was dann aber wieder Einfluss auf Medikamentendeposition hätte [6].

Der Aerosolausstoß der Mesh-Vernebler wurde vom Flow hingegen kaum beeinflusst, was für den Rettungsdienst abermals vorteilhaft wäre. Die Vernebler können zudem problemlos mit einem zusätzlichen Sauerstoffreservoir betrieben werden, was eine exakte Anpassung des Sauerstoffbedarfs an die Bedürfnisse von hypoxischen Patient*innen parallel zur Verneblung erlauben würde.

### Salbutamol-Filterdeposition

Die vorliegende Studie konnte zeigen, dass eine hohe Verneblerleistung zu einer hohen Salbutamol-Filterdeposition führt. Zwar konnte der Jet-Vernebler durch Verdopplung des Aerosolausstoßes seine Deposition verdreifachen, im direkten Vergleich mit dem M‑Neb mobile war die Filterdeposition bei einer Sauerstoffflussrate von 6 l/min allerdings 9‑mal, bei einem Flow von 12 l/min immer noch 2,5-mal geringer [21].

Was bedeutet dies für die klinische Praxis? Bei einem Flow von 6 l/min, wie er im Rettungsdienst routinemäßig zum Betrieb eines Jet-Verneblers verwendet wird, erreichten die getesteten Mesh-Vernebler die Gesamtmenge des Salbutamols, die der Jet-Vernebler innerhalb von 10 min deponiert, bereits nach 1:04 min (M-Neb) bzw. nach 1:17 min (Aeroneb). Im vorliegenden In-vitro-Modell nutzten wir zur besseren Analytik eine 2,5-mg/ml-Salbutamollösung. Die normalerweise im Rettungsdienst verwendete Lösung hat eine deutlich niedrigere Konzentration (0,5 mg/ml). Um die gleiche Deposition wie im Modell zu erreichen, hätte der Jet-Vernebler 5‑mal länger, also fast 50 min, vernebeln müssen, die Mesh-Vernebler hingegen würden lediglich etwas mehr als 5 min benötigen.

Die Medikamentendeposition kann unter normalen Umständen durch die Verwendung eines Spacers verbessert werden [3]. Der von uns getestete Spacer führte allerdings zu keiner Erhöhung der Deposition, weil aufgrund der schlechten Dichtigkeit einer normalen Gesichtsmaske bei der Inspiration nicht genügend Unterdruck erzeugt wurde, um das Aerosol wirksam aus dem Spacer zu mobilisieren.

Das Atemmuster hingegen hatte großen Einfluss auf die Salbutamoldeposition. Bennett et al. zeigten bereits die Bedeutung der Atemfrequenz und des I:E-Verhältnisses [7]. Vor allem die Länge der Inspirationszeit ist wichtig, da auch während der Ausatmung kontinuierlich Aerosol produziert wird, welches dann in die Umgebung verloren geht. Auch bei uns wurde die höchste Deposition bei den Atemmustern gefunden, die durch eine erhöhte Atemfrequenz und eine verlängerte Inspirationszeit gekennzeichnet waren. Diese Ergebnisse suggerieren, dass bei der prähospitalen Aerosoltherapie v. a. bei schwer symptomatischen Patient*innen, bei denen das Erreichen eines hohen Wirkstoffspiegels wünschenswert ist, auch die höchste Medikamentendeposition erreicht werden könnte. Dennoch sollte im Hinterkopf behalten werden, dass in Abhängigkeit des Atemmusters, z. B. im Rahmen einer Bradypnoe, eine Unterdosierung der Medikamente erfolgen könnte.

### Klinische Bedeutung der Ergebnisse

Schon seit vielen Jahren wird versucht, die Aerosoltherapie von Notfallpatient*innen zu verbessern. Der Vergleich von konventionellen Jet-Verneblern mit damals neu entwickelten Jet-Verneblern, die nur bei der Einatmung Aerosol abgeben („breath-actuated jet nebulizer“), zeigte eine in vitro nachgewiesene Depositionserhöhung von 14,4 auf 34,2 %. Darauffolgende Studien in Notfallambulanzen kamen jedoch zu sehr heterogenen Ergebnissen ohne deutliche Tendenz zur Verbesserung des Patienten-Outcomes [4, 20, 25, 26, 28]. Eine Verbesserung des Outcome konnte erstmals beim direkten Vergleich von Jet- und modernen Mesh-Verneblern nachgewiesen werden, wobei es sich hier um 2 „Single-center“-Studien mit einer relativ kleinen Fallzahl handelt [13, 17]. Ursächlich für das verbesserte Outcome könnten neben der sowohl in vitro als auch in vivo gefundenen besseren Verneblungseffizienz auch die höheren Depositionsraten mit Spitzenwerten von bis zu 44 % sein, allerdings wären groß angelegte Multizenterstudien wünschenswert, um die Bedeutung des Effektes zu untersuchen [11, 22].

Die in der vorliegenden Studie gefundenen Ergebnisse suggerieren eine Überlegenheit der Mesh-Verneblern im prähospitalen Setting. Ob die höhere Verneblereffizienz und die höhere Medikamentendeposition pro Zeit auch einen positiven therapeutischen Effekt auf Notfallpatient*innen in vivo haben, kann nur durch klinische Studien im Rettungsdienst beantwortet werden. Allerdings implizieren die genannten klinischen Untersuchungen aus den Notfallambulanzen, dass sich weitere Untersuchungen auf diesem Feld lohnen könnten.

### Limitationen

Studien konnten zeigen, dass die Deposition von Aerosolen in der Lunge in vitro im Vergleich zu in vivo überschätzt wird [9, 23]. Doch selbst wenn im vorliegenden In-vitro-Modell die Medikamentendeposition in der Lunge überschätzt wird, so sollte v. a. die Depositionsleistung des in der Praxis routinemäßig verwendeten Jet-Verneblers alarmierend sein, da sie in vivo vermutlich noch schlechter ist.

In der vorliegenden Arbeit wurde aus analytischen Gründen des fotometrischen Salbutamolnachweises eine 2,5-mg/ml-Salbutamollösung verwendet. Diese Konzentration lag somit um das Fünffache höher als bei der klinisch verwendeten Salbutamollösung (0,5 mg/ml), wodurch die gemessene absolute Salbutamoldeposition die klinisch zu erwartende übertrifft. Das Verhältnis der Medikamentendeposition der verschiedenen Vernebler bleibt aber unabhängig von der verwendeten Lösung bestehen, da die Mesh-Vernebler auch bei niedrigeren Konzentrationen durch den erhöhten Output ein Vielfaches des Jet-Verneblers deponieren.

In der Einzelanwendung sind Jet-Vernebler allgemein günstiger (Intersurgical® Cirrus™2 als Einzelartikel ca. 1,50 €, Stand April 2022). Mesh-Vernebler sind wesentlich teurer: Der (einmalige) Anschaffungspreis des notwendigen Controllers kostet je nach Modell und Hersteller etwa zwischen 350 € und 900 €. Außerdem wird patientenindividuell eine Verneblerkammer benötigt, die etwa 40–60 € kostet. Die Betrachtung einer Kosten-Nutzen-Erwägung ist allerdings komplex: Durch eine verkürzte Behandlungsdauer in der Notfallambulanz oder verminderte Hospitalisierungs- und Beatmungsraten, wie in den Studien von Cushen und Dunne angedeutet, ist eine langfristige Kostenreduktion denkbar [13, 17]. In die Überlegung der Kosteneffizienz sollten außerdem auch ein möglicherweise besseres Outcome und eine bessere Lebensqualität der Patient*innen einbezogen werden („quality-adjusted life years“ [QALY]). Die Kosteneffizienz unter Einbeziehung dieser und anderer Faktoren sollte nach Vorliegen umfangreicher klinischer Studien bewertet werden.

Alle in der vorliegenden Arbeit getesteten Vernebler wurden nach den Kriterien für einen Einsatz im Rettungsdienst ausgewählt. Die Verwendung des Jet-Verneblers ist prähospitaler Standard. Nachteile sind der obligat notwendige Frischgasfluss sowie die deutliche Geräuschentwicklung beim Vernebeln. Auch darf das Mindestfüllvolumen nicht unterschritten werden, und die Position der Verneblerkammer muss engmaschig kontrolliert werden, da der Jet-Vernebler sonst nicht effizient arbeitet. Die Mesh-Vernebler erfüllen ebenfalls alle Anforderungen an eine mobile Nutzung. Sie arbeiten annähernd geräuschlos, der Umgang ist ebenfalls unkompliziert und schnell erlernbar. Ein Nachteil ist das obligate Vorhandensein einer Stromquelle (Powerbank für Aerogen® Solo bzw. Lademöglichkeit für den integrierten Akku für M‑Neb® mobile).

## Fazit für die Praxis

- Die vorliegende Studie zeigt, dass die Verwendung von Mesh-Verneblern die Aerosoltherapie bei simulierten prähospitalen Notfallpatient*innen verbessern kann, da sie Jet-Verneblern in Bezug auf Verneblungsleistung und Medikamentendeposition überlegen sind.
- Die Verneblungsleistung war für die Gesamtdeposition des Salbutamols in der simulierten Lunge entscheidend, da sich die Deposition auch durch die Verwendung eines Spacers nicht verbessern ließ. Eine hohe Atemfrequenz war mit einer hohen Medikamentendeposition vergesellschaftet, was klinisch bei der Behandlung von schwer dyspnoischen Patient*innen gewünscht ist. Im Umkehrschluss muss aber auch mit einer Medikamentenunterdosierung bei bradypnoischen Patient*innen gerechnet werden.
- Die Höhe des supplementären Sauerstoffflusses hatte großen Einfluss auf die Leistung des Jet-Verneblers, aber so gut wie keinen Einfluss auf die Leistung der getesteten Mesh-Vernebler.
- Klinische Studien müssen zeigen, ob diese Erkenntnisse auch Relevanz für die Therapie in vivo haben.

## Supplementary Information




