# Supplementary material for: Can mesh nebulizers improve prehospital aerosol therapy? An in vitro study on simulated prehospital emergency patients suffering from respiratory distress
Source: Anaesthesiologie. 2022 Aug 17;71(10):758–66. [Article in German] doi: 10.1007/s00101-022-01183-y (PMC9525251; doi:10.1007/s00101-022-01183-y)
Supplement: Supplementary file 1 [file 101_2022_1183_MOESM1_ESM.pdf]

Zusatzmaterial zum Beitrag „Können Mesh-Vernebler die prähospitale Aerosoltherapie verbessern? Eine in vitro Studie an simulierten Notfallpatient*innen mit Atemnot“ von Otto M, Kropp Y, Kummer L et al. (2022) in *Die Anaesthesiologie*
Beitrag und Zusatzmaterial stehen Ihnen auf www.springermedizin.de zur Verfügung. Bitte geben Sie dort den Beitragstitel in die Suche ein.

Tabellen des Elektronischen Zusatzmaterials

Tab. A
Verneblerleistung (Aerosolausstoß in 10 Minuten) der drei getesteten Vernebler nach einer Verneblungszeit von 10 Minuten bei zwei bzw. drei unterschiedlichen Sauerstoffflussraten

		Verneblerleistung (ml/10 min)		
	**Vernebler**	1 l O_2/min	6 l O_2/min	12 l O_2/min
**Jet**	Cirrus 2 (a) (n = 40)	n/a	**1.05 ± 0.06**<br>< .001[b]<br>< .001[c]<br>< .001[d]	**2.36 ± 0.17**<br>< .001[b]<br>< .001[c]<br>< .001[d]
**Mesh**	Aerogen Solo ohne Spacer (b) (n = 60)	**3.60 ± 0.26**<br>< .001[c]<br>< .001[d]	**3.99 ± 0.3**<br>< .001[a]<br>> .99[c]<br>< .001[d]	**3.99 ± 0.17**<br>< .001[a]<br>> .99[c]<br>< .001[d]
	Aerogen Solo mit Spacer (c) (n = 60)	**3.83 ± 0.24**<br>< .001[b]<br>< .001[d]	**4.00 ± 0.34**<br>< .001[a]<br>> .99[b]<br>< .001[d]	**3.96 ± 0.21**<br>< .001[a]<br>> .99[b]<br>< .001[d]
	M-Neb mobile (d) (n = 60)	**4.7 ± 0.75**<br>< .001[b]<br>.002[c]	**5.23 ± 0.64**<br>< .001[a]<br>< .001[b]<br>< .001[c]	**5.47 ± 0.87**<br>< .001[a]<br>< .001[b]<br>< .001[c]

[a] P verglichen mit der Leistung des (a) Cirrus 2 bei gleichem Sauerstofffluss
[b] P verglichen mit der Leistung des (b) Aerogen Solo (ohne Spacer) bei gleichem Sauerstofffluss
[c] P verglichen mit der Leistung des (c) Aerogen Solo (mit Spacer) bei gleichem Sauerstofffluss
[d] P verglichen mit der Leistung des (d) M-Neb mobile bei gleichem Sauerstofffluss

Tab. B
Salbutamoldeposition der drei getesteten Vernebler nach einer Verneblungszeit von 10 Minuten bei zwei bzw. drei unterschiedlichen Sauerstoffflussraten

		Salbutamoldeposition (mg)		
	**Vernebler**	1 l O_2/min	6 l O_2/min	12 l O_2/min
**Jet**	Cirrus 2 (a)<br>(n = 40)	n/a	**0.36 ± 0.09**<br>< .001[b]<br>< .001[c]<br>< .001[d]	**1.22 ± 0.34**<br>< .001[b]<br>< .001[c]<br>< .001[d]
**Mesh**	Aerogen Solo ohne Spacer (b)<br>(n = 60)	**1.82 ± 0.41**<br>< .001[c]<br>0.025[d]	**2.82 ± 1.0**<br>< .001[a]<br>> .99[c]<br>0.658[d]	**2.50 ± 0.17**<br>< .001[a]<br>> .99[c]<br>< .001[d]
	Aerogen Solo mit Spacer (c)<br>(n = 60)	**1.76 ± 0.65**<br>.19[b]<br>.002[d]	**2.80 ± 0.78**<br>< .001[a]<br>> .99[b]<br>< .001[d]	**2.55 ± 0.9**<br>< 0.001[a]<br>> .99[b]<br>0.284[d]
	M-Neb mobile (d)<br>(n = 60)	**2.47 ± 0.68**<br>.025[b]<br>.046[c]	**3.36 ± 0.64**<br>< 0.001[a]<br>.66[b]<br>.40[c]	**3.21 ± 0.97**<br>< .001[a]<br>.28[b]<br>.51[c]

[a] P verglichen mit der Deposition des (a) Cirrus 2 bei gleichem Sauerstofffluss
[b] P verglichen mit der Deposition des (b) Aerogen Solo (ohne Spacer) bei gleichem Sauerstofffluss
[c] P verglichen mit der Deposition des (c) Aerogen Solo (mit Spacer) bei gleichem Sauerstofffluss
[d] P verglichen mit der Deposition des (d) M-Neb mobile bei gleichem Sauerstofffluss
